# Importance of Standardizing Analytical Characterization Methodology for Improved Reliability of the Nanomedicine Literature

**DOI:** 10.1007/s40820-022-00922-5

**Published:** 2022-08-20

**Authors:** Shahriar Sharifi, Nouf N. Mahmoud, Elizabeth Voke, Markita P. Landry, Morteza Mahmoudi

**Affiliations:** 1 Department of Radiology and Precision Health Program, Michigan State University, East Lansing, MI USA; 2 Faculty of Pharmacy, Al-Zaytoonah University of Jordan, Amman, 11733 Jordan; 3 Department of Biomedical Sciences, College of Health Sciences, QU Health, Qatar University, 2713 Doha, Qatar; 4 Department of Chemical and Biomolecular Engineering, University of California, Berkeley, Berkeley, CA USA; 5 Innovative Genomics Institute, Berkeley, CA USA; 6 California Institute for Quantitative Biosciences, University of California, Berkeley, CA USA; 7 Chan Zuckerberg Biohub, San Francisco, CA USA

**Keywords:** Characterization, Nanomedicine, Standard protocols, Reproducibility, Nanomedicine devices

## Abstract

Understanding the interaction between biological structures and nanoscale technologies, dubbed the nano-bio interface, is required for successful development of safe and efficient nanomedicine products. The lack of a universal reporting system and decentralized methodologies for nanomaterial characterization have resulted in a low degree of reliability and reproducibility in the nanomedicine literature. As such, there is a strong need to establish a characterization system to support the reproducibility of nanoscience data particularly for studies seeking clinical translation. Here, we discuss the existing key standards for addressing robust characterization of nanomaterials based on their intended use in medical devices or as pharmaceuticals. We also discuss the challenges surrounding implementation of such standard protocols and their implication for translation of nanotechnology into clinical practice. We, however, emphasize that practical implementation of standard protocols in experimental laboratories requires long-term planning through integration of stakeholders including institutions and funding agencies.

## Highlights

- The use of current standard protocols for robust characterization of nanotechnologies can significantly improve the reproducibility of nanoscience data particularly for studies seeking clinical translation.
- The use of current standard protocols for robust characterization of nanotechnologies can significantly improve the reproducibility of nanoscience data particularly for studies seeking clinical translation.
- Institutions, funding agencies, and publishing venues have a vital role in the practical implementation of the standard protocols of nanomaterials characterization.

## Graphical abstract

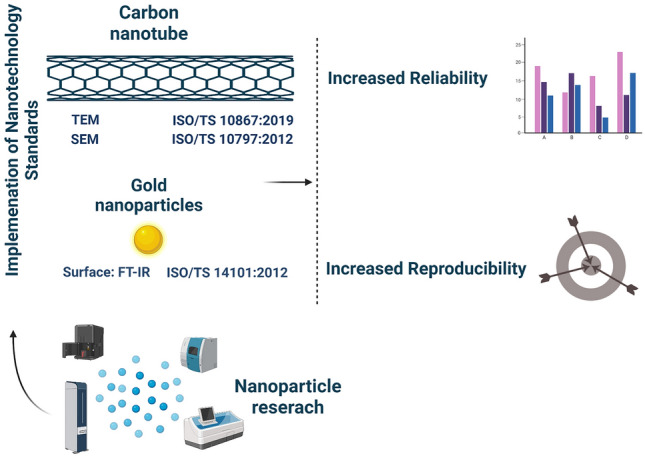

## Introduction

Nanomedicine is an umbrella term defined by the Encyclopedia Britannica as “a branch of medicine that seeks to apply nanotechnology—that is, the manipulation and manufacture of materials and devices that are roughly 1 to 100 nm (nm; 1 nm = 0.0000001 cm) in size—to the prevention of disease and to imaging, diagnosis, monitoring, treatment, repair, and regeneration of biological systems” [1]. According to this definition, it is clear that nanomedicine covers a broad range of medical products ranging in purpose and performance requirements from therapeutic nanoparticles containing drugs to be delivered *in vivo* with stringent control to nano-biosensors for development of in vitro/ex vivo diagnostics kits with lower associated risk compared to injectable nanoformulations [2–5]. In general, nanomedicine has shown significant therapeutic success in both in vitro and non-human *in vivo* studies. However, successful clinical translation of nanomedicines has lagged behind the successes implied by numerous positive preclinical findings [6–8]. Unlike the *in vitro* conditions in which most nanotechnologies are validated, when nanoscale materials enter biological environments, their physiochemical properties change through the spontaneous adsorption and interaction of the synthetic nanoscale material with the complex biomolecular environment, forming the nano-bio interface. This nano-bio interface is incredibly complex, dynamic, and difficult to characterize. While scientific reproducibility is an issue that plagues multiple fields of study [9], the unique multidisciplinary nature of nanomedicine may contribute to challenges of methodological reliability and reproducibility issues. Nanomedicine combines expertise from several fields including materials science, chemistry, biology, physics, pharmacology, and the like, and it can therefore be particularly challenging to establish best practices for collecting and reporting data when working across multiple fields.

One can assume that if unreliability or lack of reproducibility [10–12] were the major barriers in nanomedicine, few products would pass through the regulatory pipeline to reach the market. However, an increasingly large number of medical products using nanotechnology have reached the market, comprising a multi-billion dollar industry, such as the recently developed SARS-CoV2 vaccine formulations by Moderna and Pfizer/BioNTech among others [13]. More generally, according to the FDA database of medical devices, about 2586 different 'nano' medical devices were sold in the USA from 1980 to 2017 [14]. These nanomedical devices have mainly included in vitro diagnostic devices including nanosilver or nanogold particles used ubiquitously in *ex vivo* tests such as rapid at-home tests, orthopedic and dental implants with nanostructure surfaces, wound dressings including silver nanoparticles or nanofibers, bone void fillers including nano-calcium phosphates, stents including nanomaterial coating, vascular grafts including nanomaterial coatings, or catheters coated with silver or other nanomaterials [15]. Conversely, a few of these nanomedical devices consist of nanoparticulate formulations used for parenteral and intravenous administration [16]. The list of commercial FDA-approved nano-drugs, in which drugs are encapsulated for delivery by nanoparticles, is quite short and limited to a few well-established nanoscale systems such as lipid nanoparticles [17]. Herein, we describe the challenges to creating “blanket” analytical and reporting guidelines for nanomaterials and nanomedicine research, the consequences thereof, and some of the initiatives that have begun to take hold in the field to establish standard guidelines.

## Causes and Solutions to Reproducibility in Nanomaterials Research and Reporting

Several factors can contribute to poor methodological quality and reproducibility of nanoscience publications, independent of whether those nanotechnologies are subsequently used in biomedical applications. Additionally, the “file drawer” issue (i.e., the tendency to only publish the positive results) in nanomedicine may misrepresent actual findings and remains under-investigated [18]. More broadly, a lack of nanoparticle characterization with a universally established quality control pipeline is considered a primary challenge to building a cohesive body of literature in nanotechnology and makes it particularly challenging to predict how these nanotechnologies will fare in biological environments. For instance, it is common to assess nanomaterial properties such as the chemical composition, polydispersity index, size, charge, and other such physiochemical properties in vitro despite the intended use of nanotechnologies in vivo*.* Similarly, there is a lack of standardization for what physiochemical properties should be assessed, and whether the reported variabilities represent average measurements over multiple experimental replicates of the same sample (less rigorous) versus multiple technical replicates over independently synthesized nanoparticle batches (more rigorous). Another common issue is that solution-phase characterization methods such as dynamic light scattering (DLS) are difficult to implement for non-spherical nanoparticles or highly polydisperse samples. For example, studies revealed significant variations in the DLS results of the exact same type of nanoparticles through different laboratories [19]. This lack of universally established quality controls likely contribute to variable reproducibility outcomes in nanoscience. These lacks of standardized guidelines are particularly deleterious for nanoscience studies wishing to implement nanotechnology in biomedicine. Another common theme in nanomaterials research is a lack of thorough analytical validation of nanoparticle surface chemistry. For instance, carbon nanotubes (and numerous other types of nanoparticles) have been the subject of a decades-long debate on their biocompatibility, or lack thereof [20]. However, many studies, particularly those claiming cytotoxicity, lack analytical validation of nanoparticle purity and/or surface chemistry [21]. Meanwhile, nanomaterial purity can vary greatly from the specifications provided by the manufacturer, such as commercially-procured carboxylated nanotubes (COOH-SWNT) that were found to contain over 80% more amorphous carbon contamination than specified by its supplier, Sigma-Aldrich [22]. Therefore, it is possible that reports of carbon nanotube toxicity may originate from the toxicity of residual nanotube synthesis by-products such as amorphous carbon and residual metal precursor catalysts, or from intended or unintended modifications to nanotube surface chemistry [23]. Similarly, toxicity reports of other nanomaterials may also be related to impurities or bi-products in their production, rather than the nanomaterial itself - a distinction that is not possible to determine without detailed analytical characterization of the studied nanomaterial samples. 

**Fig. 1:**
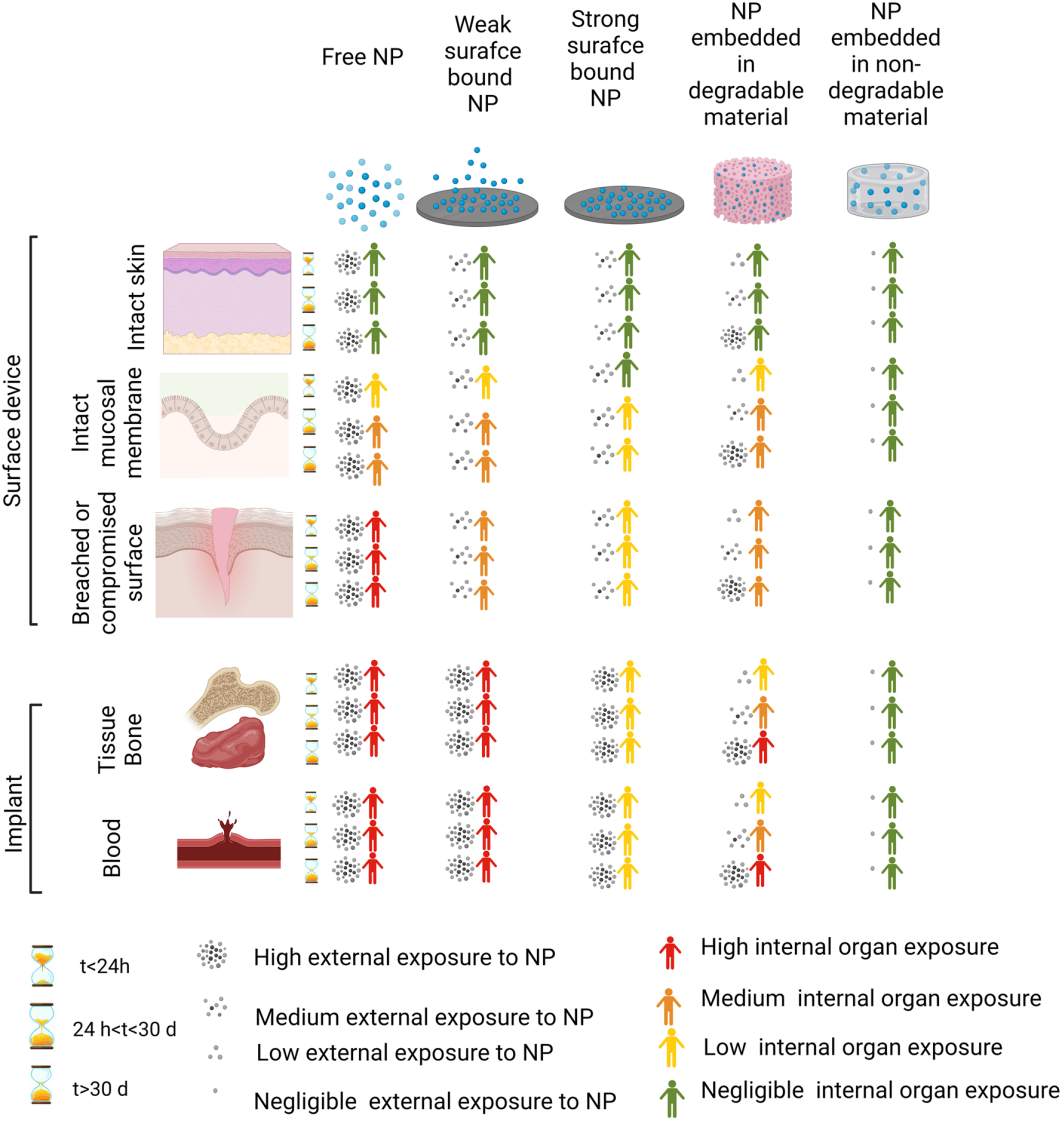
Risk evaluation of nanomedicine devices based on estimation of internal and external exposure. Based on the type of organ, duration of exposure of nanomaterials, and their physicochemical characteristics, humans can be subject to negligible or high internal exposure to nanomaterials. The figure is drawn based on the data provided in the Scientific Committee on Emerging and Newly Identified Health Risks (SCENIHR) report entitled “Guidance on the determination of potential health effects of nanomaterials used in medical devices” [25]

Generally, it is common to see that the rigor and extent of nanoparticle characterization is directly proportional to the risk associated with the downstream use of that nanotechnology in biomedical applications. For example, the Scientific Committee on Emerging and Newly Identified Health Risks (SCENIHR) recommends a risk-based system in which stringency of evaluation and characterization of nanomaterials depends on nanomaterial type, duration of contact with the body, and the nature of patient interaction with devices (Fig. 1) [24]. For instance, known bio-incompatible materials that would interact directly with human tissues and/or are likely to leak out of their devices during use would be subject to more stringent characterization and biocompatibility testing than nanomaterials that are known to be biocompatible and/or are unlikely to come into direct contact with patients in clinical use. The characterization of nanomaterials in scientific papers, however, can vary widely from study to study since these materials are mostly used in preclinical animal models. One common omission from many academic studies is analytical assessments of nanomaterials when used as-procured from commercial sources, despite large possible variabilities in the quality and purity of commercially procured nanomaterials from supplier-to-supplier, from batch-to-batch, and even from the vendor-provided spec sheet [22]. Efforts to increase the rigor and reproducibility of nanomaterials science research have been initiated both from the scientific community and from national regulatory bodies as described below.

## Research Community-driven Efforts to Increase Rigor and Reproducibility of Nanoscience Studies

To improve the reproducibility of nanoscience-based publications intended for use in biomedical applications, Caruso and co-workers recently proposed the use of a reporting checklist, Minimum Information Reporting in Bio-Nano Experimental Literature (MIRIBEL), as a requirement for publication [21]. MIRIBEL checklist items include, for example, details on material characterization (e.g., synthesis, composition, size, shape, dimensions, size dispersity, and aggregation), biological characterization (e.g., cell seeding details, cell characterization, and passage), and experimental protocol details (e.g., culture dimensions, administered dose, method of administration, and delivered dose) [21]. Expert responses from the nanomedicine community, despite supporting the initial goals of the MIRIBEL checklist, demonstrated the need for a list more closely tailored and continuously adjusted to criteria based on the intended use of nanomedicines [10, 26]. In addition to the checklist, collecting accurate and valid information on the characterization of nanomedicines using standardized methodologies is a key step toward a more precise understanding and/or prediction of the safety and therapeutic/diagnostic efficacies of nanomedicine products. In other words, although reporting minimum information related to type and results of characterization is essential that is insufficient to guarantee the repeatability and reliability of nanomedicine data. For instance, many characterization techniques such as DLS or zeta potential measurements are highly dependent on experimental conditions and sample preparation details. Therefore, reporting a value of physicochemical properties (e.g., size via DLS) without mentioning data acquisition details (e.g., for DLS: type of medium employed, concentration of particles, type or size of cuvette, wavelength of laser, filtration conditions, and data rendering: number count vs. raw intensity) may result in discrepancies between laboratories on identical nanoparticles. For example, it was shown that the outcomes of two of the most commonly applied techniques for nanoparticle sizing (i.e., DLS and differential centrifugal sedimentation) of identical nanoparticles by different laboratories were significantly different [19].

## Nationally driven Efforts to Increase Rigor and Reproducibility of Nanoscience Studies

On a national and international scale, regulatory agencies in the USA and Europe have issued and established several sets of rules, guidelines, and recommendations for evaluation of nanomedicine products. For example, the FDA evaluates nanomedicinal products on a case-by-case basis, employing the combination product framework to determine the type of product and resulting regulatory requirements for its review [5]. The FDA has identified several challenges and gaps related to physicochemical characterization, biocompatibility, and toxicity to evaluate nanoparticles that are incorporated into medical devices and consequently has launched the Nanotechnology Program in the FDA's Center for Devices and Radiological Health (CDRH). The program focuses on regulatory research in evaluating the physicochemical properties and toxicity of nanomaterials utilized in medical devices, and the impact of the manufacturing processes on these properties. Similarly, EU regulation such as 2017/745 specifically recommends highly stringent conformity assessment procedures (Class III, highest class risk in Europe) for medical devices containing nanomaterials, particularly when nanomaterials in those devices carry a high likelihood of direct human exposure.

Considering these regulations and standards more closely, standards such as ISO/IEC17025 “testing and calibration laboratories” or good laboratory practice (GLP) as defined by either the FDA or the European Council evaluate an organization’s technical competence in using analytical instruments or testing to generate reliable and reproducible results [27]. Implementation of GLP requires establishing a quality assurance team to verify and document compliance with GLP rules, verification of the maintenance and calibration of analytical instruments, validation of analytical methods, as well as development of standard operation procedures. Academic settings or laboratories seldom implement all such standards or regulations, likely due to a dearth of training on quality- or GLP-related subjects [28]. It seems that GLP and similar regulations such as GMP are ultimately established to protect patient health and safety; the end-users of nanoscale medical biotechnologies. However, the implementation of these regulations in the academic research settings is limited. Academic laboratories currently emphasize safe standard operating procedures implemented in the laboratory setting with a center on experimenter and environmental health and safety. However, implementation of GLP regulations that focus on rigor and reproducibility of nanomedical devices in clinical setting in academic research settings is not mandatory, since most academic research studies are not directly connected to patient trials or clinical health outcomes. However, this lack of universal quality control in academic settings can make it difficult to identify nanotechnologies that are the most promising pre-clinical candidates and can lead to premature in vivo testing of sub-optimal nanomedicines or, conversely, lead to overlooking promising nanotechnologies for in vivo translation.

## Non-governmental Organization Standards and Guidelines for Nanomaterial Characterization

**Fig. 2:**
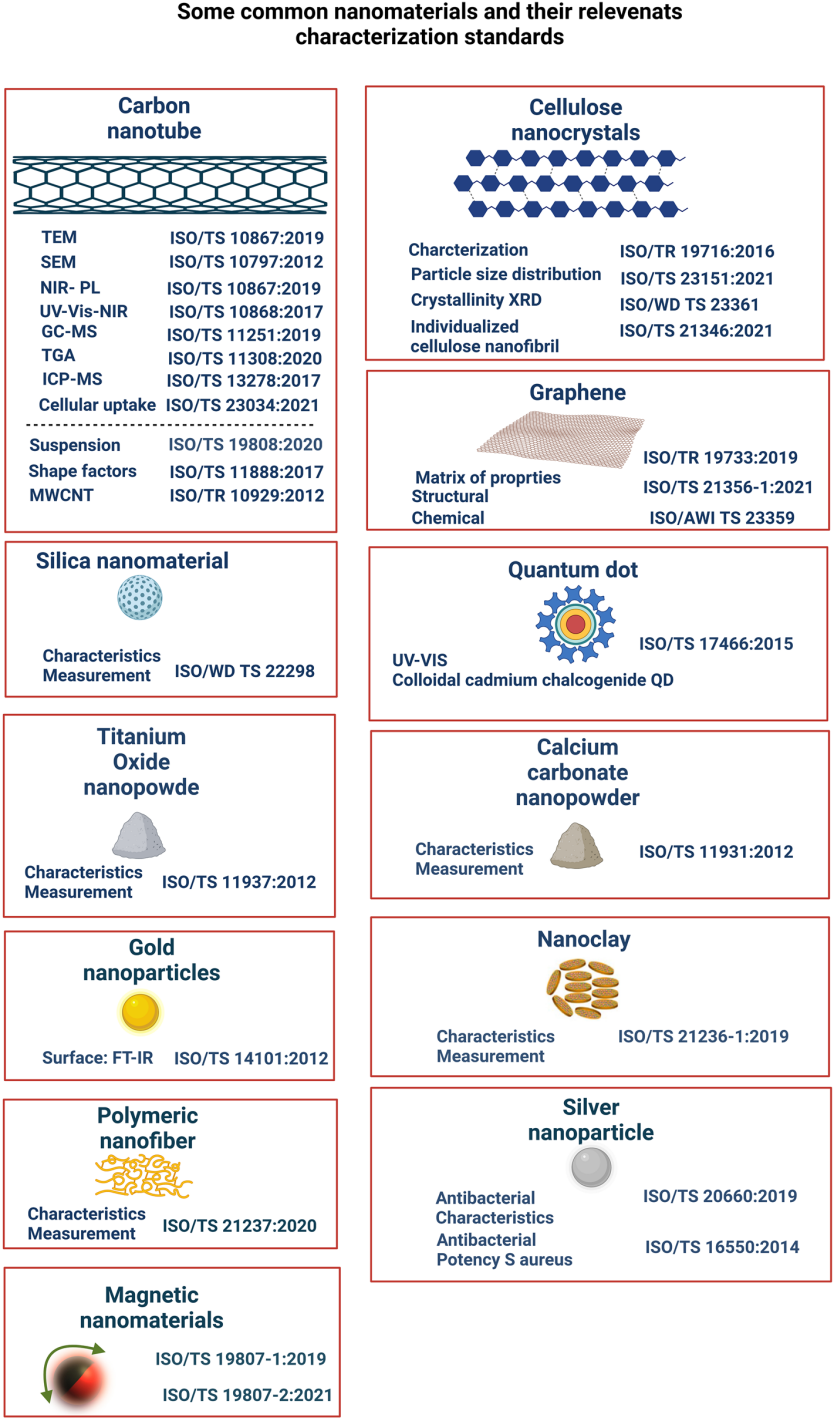
Examples of available material-specific standards and characterization methodologies for different nanomaterials. Documentation on characterization methodologies for different nanomaterials is available based on ISO ID numbers and can help compile best practices for the analysis and characterization of different nanomaterials

Standards institutes and regulation agencies have provided a number of documents and guidelines to address the requirements of analytical characterization and validation of nanomaterials. For example, one of the sets of available standards for nanomaterial characterization was provided by the International Organization for Standardization (ISO; www.iso.org). ISO is a nongovernment organization and network of national standards institutes in 162 countries, represented by one member in each country. The ISO Technical Committee (TC) 229 was established in 2005 standardize the process of creating and reporting new nanoscale materials. The ISO (TC) 229 committee has five working groups designed to develop standards for terminology and nomenclature, measurement and characterization, health and safety, materials specification as well as product and application of nanomaterials [29, 30]. Until now, the ISO (TC) 229 committee has published 100 standards related to nanotechnology, with 31 still under development. The material-specific standards in the ISO (TC) 229 series are listed in Fig. 2 which notably lists several characterization methods for each class of nanomaterial. In addition to material-specific standards (Fig. 2), the ISO and especially the ISO (TC) 229 have also developed other standards for robust characterization of nanomaterials. For example, scanning electron microscope (SEM) and transmission electron microscope (TEM) imaging are the gold standards for characterizing the shape, size, and size distribution of nanoparticles. The International Standard ISO 19749 and ISO 21363 provide guidelines related to sample preparation for powder and liquid samples, deposition of nanoparticles on a substrate, number of samples to be prepared and measured, number of particles to be measured for particle size and shape determination, image acquisition, data analysis, and reporting of results. The standards report that uniform distribution of nanoparticles with minimal aggregation across the entire measurement substrate is essential for accurate analysis and reporting of particle shape, size, and size distribution. Measurement errors can arise with aggregated particles, such as aggregated particles being counted as one particle. The current standards present essential sample preparation techniques such as powder and liquid sample deposition techniques with minimum agglomeration, selecting a suitable measurement substrate that enhances the contrast between particle and background, and the adhesion of the particles across the substrate, optimizing the concentration of the sample, drying methods, and use of representative samples. It is important to note that these optimizations are often user-defined and thus subject to human biases. Furthermore, the quality of TEM and SEM images are strongly dependent on several factors including sample preparation, nanoparticle size, nanoparticle stability under electron beam exposure, and nanomaterial atomic mass.

## Efforts Implemented to Increase Nanomaterials Science Reproducibility

The establishment of above-detailed standards represent an important step forward for the nanoscience community yet implementing these standards and enforcing their use for publication remains sporadic. Currently, thorough characterization of nanomaterials using several orthogonal analytical techniques is considered best practice in the nanoscience community. Numerous techniques are often available to analyze each physiochemical property, and selection of which analytical techniques used to characterize nanoparticle samples are often based on their eventual intended use in nanomedicine [11]. Furthermore, an important component of GLP requirements includes ensuring that analytical methods are properly calibrated to ensure that their performance meets the requirements for the intended applications. According to USP (U.S. Pharmacopeia), the analytical characteristics of a method including accuracy, precision, specificity, detection limit, quantitation limit, linearity, range, and robustness need to be identified and validated prior to sample characterization, such that different batches and altogether different samples can be compared to each other and against the same known baseline. Conversely, many published studies focus on the formulation of drug nanocarriers and demonstrate the formulation’s intended efficacy in an animal model [31, 32]. Often overlooked are the quantification of residual or precursor materials in commercially procured starting nanomaterials and aforementioned standardization of analytical calibrations [33–37]. For instance, the validation requirements for analytical methods such as high-performance liquid chromatography for pharmaceutical analysis and quantification of active pharmaceutical ingredients in the presence of nanocarriers are already established [38] and implemented for clinical studies. Nevertheless, validation for most analytical techniques used in the characterization of nanomaterials or carriers such as DLS, zeta potential, SEM, or TEM is not a common practice [39]. Hence, there is great potential risk for nonstandard or uncontrolled analytical processes to compromise the methodological quality and, consequently, the reliability of published research works especially in the field of nanomedicine.

## Existing Standards and Guidelines for Nanomaterial Biocompatibility Assessments

**Fig. 3:**
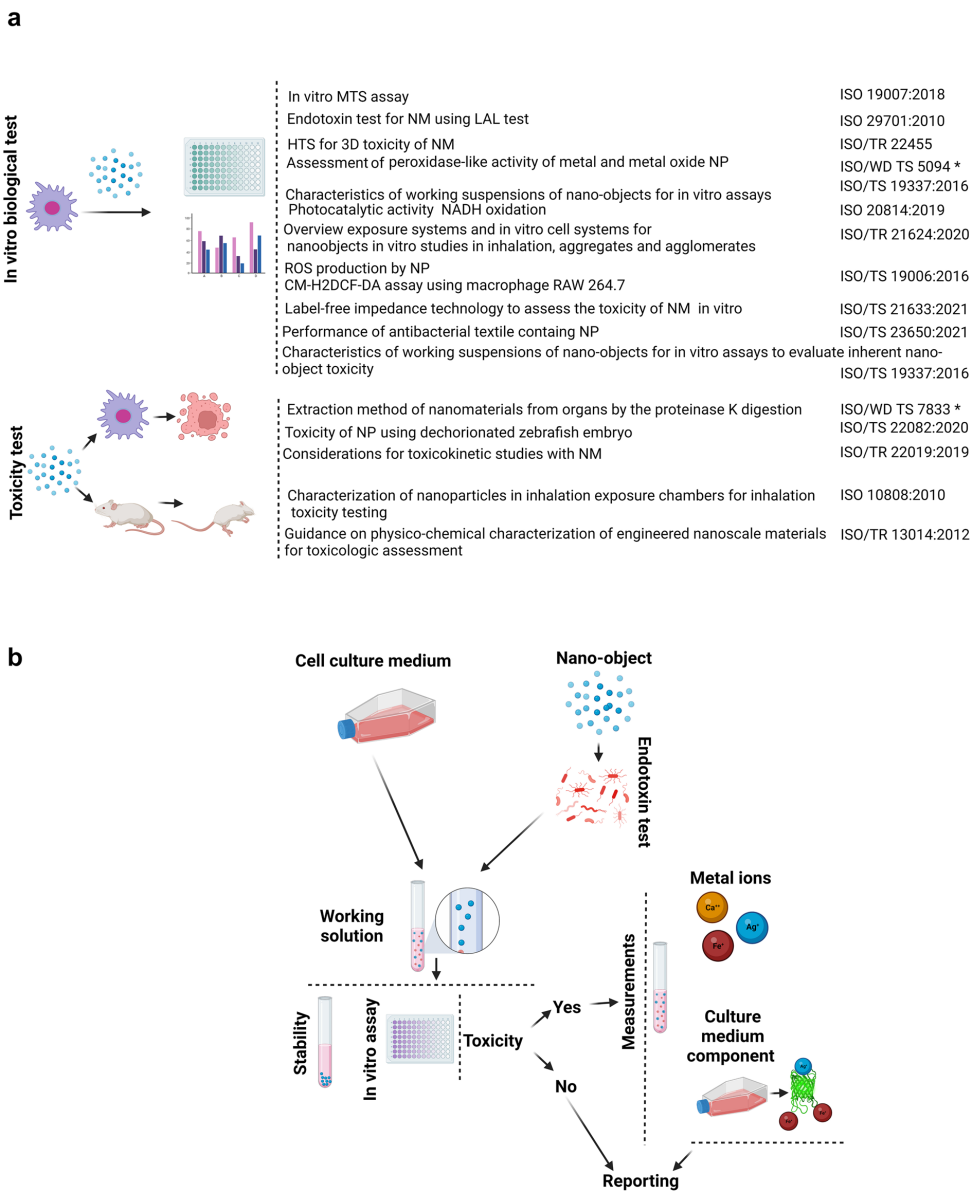
Examples of standards related to the in vitro biological tests and toxicity evaluations/monitoring of nanomaterials. **a** ISO (TC) 229 committee standards provide general guidelines and recommendations for nanoparticle characterization in biological environments, and for evaluation of nanoparticle biocompatibility in living systems. Standard labeled by “*” are currently under development. **b** Schematic showing the states at which measurements are made (Technical Specification ISO/TS 19337:2016(E))

The ISO and especially ISO (TC) 229 have also developed several standards for biological and toxicity evaluation of nanomaterials (Fig. 3a). For example, the ISO/TR 16196 and ISO/TR 16197 standards discuss essential protocols on sample preparation and dosimetry of nanomaterials for reliable toxicological screening. The Technical Specification ISO/TS 19337:2016(E) describes the characterization of working nanomaterial suspensions when conducting in vitro toxicity assays and applicable measurement methods. The technical report presents a useful staged scheme for performing measurements (Fig. 3b). For working stock suspensions of nanoparticles, the presence of endotoxins should be verified, since they may significantly alter the in vitro toxicity test results. Furthermore, other characterizations of nanoparticles that may affect the toxicity assay results should be performed and reported. For example, the working suspensions' stability, by measuring particle size and concentration as a function of time, should be assessed both in vitro and, in an environment, mimicking its intended use (i.e., in plasma if to be injected into the bloodstream). This level of analysis in biofluids is necessary because upon introduction of nanoparticles into biofluids, the surface of nanoparticles is rapidly coated with constituents of biofluids such as lipids and proteins, forming the nanoparticle protein corona [40]. Another important characterization to undertake and report is the concentration of metal ions in nanoparticle samples, where many nanomaterials are synthesized with the use of metal precursors, whose residual presence may not always be accurately reported by manufacturers but can significantly affect toxicity outcomes.

Another ISO committee, ISO/TC 194 Biological and Clinical Evaluation of Medical Devices, has also developed standards and guidelines for characterization and development of nanomaterials used in medical devices. The ISO/TC 194 committee produced the important ISO 10993–22, which compiles and disseminates the safety and biocompatibility of various nanomaterials [41]. This standard describes the characterization of nanomaterials and, in line with ISO/TR 13014, “guidelines for physicochemical characterization of nanomaterials,” provides a framework including a series of considerations and recommendations to improve the quality and reproducibility of nanomaterials’ characterization and evaluation. The ISO/TR 13014 standard recommends that some basic properties such as chemical composition, purity, object size and size distribution, aggregation and agglomeration state, shape, surface area, surface chemistry, surface charge, solubility, and dispersibility need to be assessed, and, based on the type of nanomaterial and its intended use, additional characterization such as redox potential, radical formation potential, or crystallinity may also be needed. Moreover, the ISO/TR 13014 standard also mentions specific nanomaterial characterization methods to be routinely undertaken including DLS, SEM, and zeta potential measurements. In addition, the technical report ISO/TR 10993–22 describes several aspects of cytotoxicity evaluation and compatibility of nanomaterials specifically intended to be used in conjunction with existing medical or clinical diagnostic devices. Because nanomaterials can have broad absorption properties across the electromagnetic spectrum, they can interfere with standard medical or diagnostic assays which use standard dyes or fluorophores. These additive optical interactions can lead to variable results, artifacts related to nanomaterial-dye interactions, and overlooked cytotoxicity. Therefore, appropriate controls and removal of nanoparticles by centrifugation or filtration before reading results can minimize artifacts and reduce variations in the results. Furthermore, the technical report ISO/TR 10993–22 discusses guidelines for general genotoxicity, carcinogenicity, reproductive toxicity, immunotoxicity, irritation and sensitization, and hemocompatibility related to the use of nanoparticles in clinical testing settings.

**Fig. 4:**
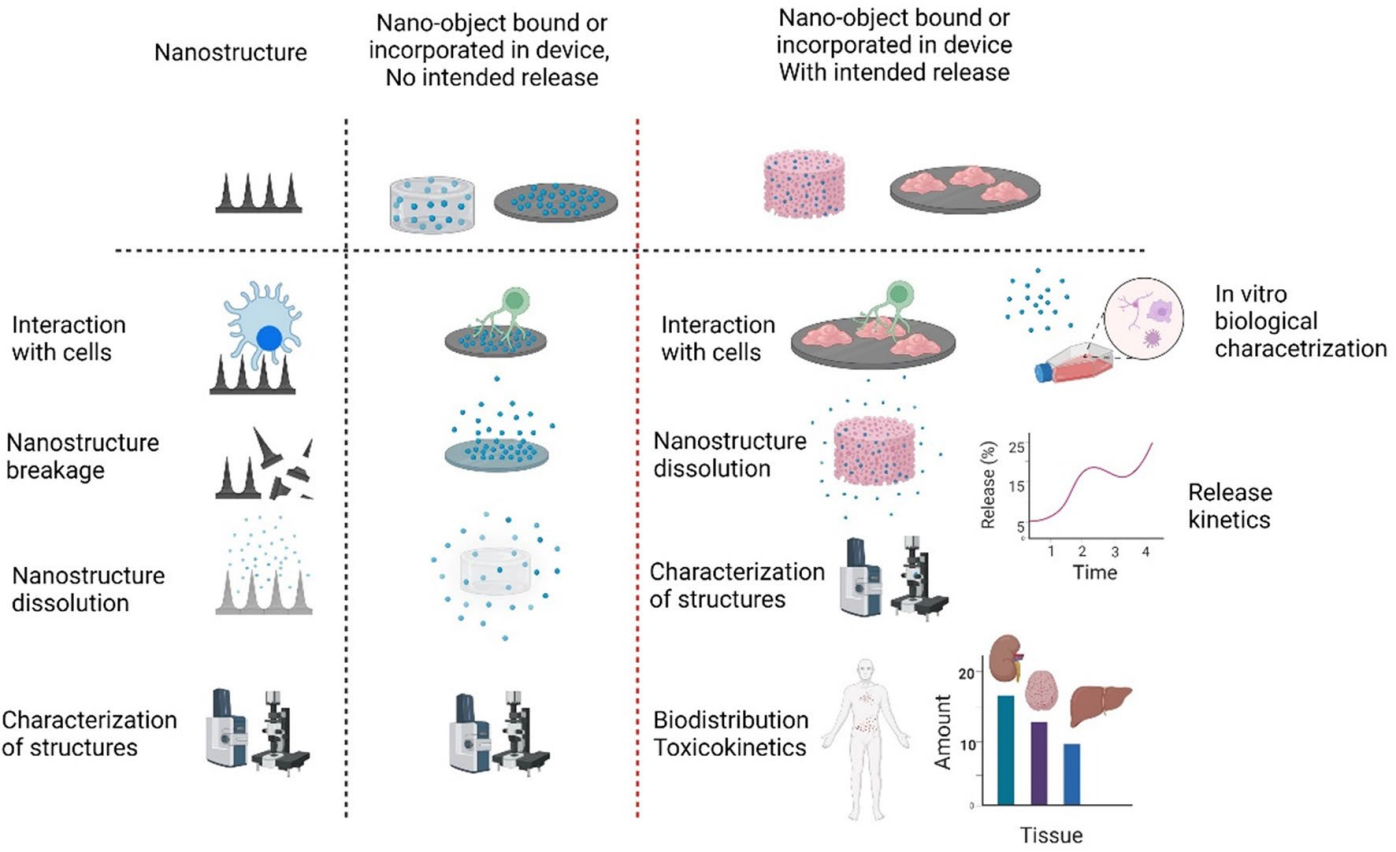
Characterization requirements for medical devices containing nanostructures and nanomaterials as recommended by ISO/TR 10993–22. The extent of characterization is dependent on the type and state of nano-based medical devices. The nanomaterial exposure risk via direct contact or unintended nanoparticle leakage from the device needs to be considered in the device characterization to properly assess safety and efficacy of nanotechnology-based medical devices. The degradation or dissolution and stability of nanostructures in relevant biological media need to be monitored and characterized over the shelf life and active lifetime of medical devices. Finally, the structures need to be fully characterized both in vitro and in in vivo proxies to ensure the design and physicochemical properties do not compromise the safety and efficacy of the medical devices. The scrutiny of the evaluation will increase if the nanostructures are designed to release from the device or pose the risk of undesired release in biological fluids. In addition to the above-mentioned evaluations, further tests (e.g., biodistribution, toxicity, and release kinetics of nanomaterials) are required to ensure the nanomedical device is safe for use in the clinic. The ISO/TR 10993–22 standards provide a framework and guidelines for characterization of nanomaterials. More specific physicochemical characteristic testing of nanomaterials is detailed in ISO/TR 13014. The figure is drawn based on the information provided in ISO/TR 10993–22 [41]

As stated above, the extent of nanomaterial characterization performed is often based on the risk associated with the product. Intended use is critical for defining the required information to be reported in the experimental literature to ensure reproducibility. For example, nanopharmaceuticals have inherently higher risk of negative biocompatibility outcomes compared to non-implantable medical devices, as their mode of action is through direct contact with a patient and can interfere with immunological, pharmaceutical, or metabolic pathways. The formulation, type of application, pharmacokinetics, as well as dosage of nanomedicine products can also define the approach to their characterization. For example, unlike nanomaterials used in pharmaceuticals, which are usually in colloidal form, nanomaterials used in medical devices can have various forms. Due to this variety in dosage and forms, the ISO/TR10993-22 also provides classifications for nanomaterials used in medical devices, either as surface-bound nanostructures, which are to be incorporated within a medical device with or without the intention of being released, versus nano-objects that might be released from a medical device as a product of degradation, versus medical devices that are themselves nanoscale objects (Fig. 4). Proper knowledge and identification of nanomaterials' physicochemical characteristics and biocompatibility prior to incorporation into medical devices are essential to understand their compatibility with other composites and determine the final product's biocompatibility and toxicological effects [42]. In addition, nanomaterials used in medical devices or nanopharmaceuticals will ultimately be exposed to biological media containing body fluids; hence, the above-mentioned parameters must include the effect of the protein corona [41]. Depending on the type of nanomaterials and whether they will be static on the device or released, the ISO/TR 10993-22 has several recommendations for safety evaluation of devices.

Based on the intended use of nano-based devices (e.g., for orthopedic implants versus ex vivo diagnostics), characterization of additional nanomaterial features should be undertaken. For example, the surface topography of implants has been shown to have critical influence in modulating the immune response as it can provoke inflammation and foreign body reactions [43]. In 2019, the FDA recalled highly textured breast implants due to risk of breast implant-associated anaplastic large cell lymphoma, a cancer of the immune system [44]. In this example, interaction of cells with these nanostructures, possibility through disintegration or breakage of these breast implant topological features, motivated standardization of nanomaterial-based implants regarding assessment of their degradation or dissolution and measurement of average surface roughness.

Although the above-mentioned standards will not cover all types of nanomaterial-based or nanomaterial-incorporated medical devices, nor all toxicity assessments, they address many critical requirements and especially characterization criteria and reporting requirements for nanomaterials used in the clinic. In addition to the surface topology example above, these standards cover various aspects of in vitro biological testing, sample preparation, and interaction of the nanomaterials with biofluids relevant to their intended use (Fig. 4). It is worth to note that demonstration of compliance to these standards is a regulatory requirement for product approval and market launch.

It is also noteworthy that standardization efforts for nanomaterial characterization are not limited to the ISO committee. The American Society for Testing and Materials (ASTM), another international standard organization, has a committee on nanotechnology (E56) which coordinates the nanotechnological need and addresses issues related to standards and guidelines related to nanotechnology and nanomaterials. ASTM E 56 has several subcommittees which include standards on informatics and terminology (E56.01), physical and chemical characterization of nanomaterials (E56.02), environment, health, and safety of nanomaterials (E56.03), nano-enabled consumer products (E56.06), education and workforce development (E56.07) and nano-enabled medical products (E56.08). The importance of training, especially regarding selection and operation of nanotechnology infrastructure, is critical as it directly affects the precision and accuracy of nanotechnology data and results. Hence, while ASTM standards cover a variety of nanotechnology characterization techniques, those standards place a particularly strong emphasis on education and training in the field. For example, ASTM E3001-20 describes a procedure for education and training on the use and analysis of characterization methods for nanometer-scale materials. The E56 and specially E56.08 also have a few guidelines and standards for medical products that have nanoscale features or use nanoparticles. For example, three standards address lipid quantification in liposomal products (E3297-21, E3323-21, and E3324-22). Similar to ISO, ASTM has also developed standards for the characterization of nanoparticles as mentioned in E56.02.

Other agencies such as the European Nanomedicine Characterization Laboratory (EUNCL) and the US National Cancer Institute Nanotechnology Characterization Laboratory (NCI-NCL) have jointly developed several standard operating procedures (SOPs) for characterization and assessment of nanomaterials used in medicine. These SOPs include size and size distribution, concentration, surface chemistry, chemical composition, in vitro assays (e.g., immunological, toxicity, oxidative stress), and in vivo assays protocols [45, 46]. The EUNCL and NCI-NCL have jointly developed multiple standard operating procedures (SOPs) for nanoparticle analyses which address method validation ranging from proper sample preparation to calibration requirements. For example, the Joint Assay Protocol, PCC-1, published by EUNCL and NCI-NCL details experimentation conditions that should be used for DLS measurements. This protocol specifies that the typical nanoparticle sample concentration for DLS measurements is 1 mg/ml, but needs to be modified according to the scattering properties of the sample. The hydrodynamic size measured by DLS is also noted to depend on the salt concentration of the suspending medium, and the guidelines recommend performing DLS measurements using supporting inert monovalent electrolytes (e.g., 10 mmol NaCl) and to avoid using pure deionized or distilled water which can cause a decrease in the measured diffusion coefficient and an apparent increase in hydrodynamic size. Furthermore, the nanomaterial sample and the dispersion medium should be filtered before measurement to prevent background scattering due to dust or contaminants. Lastly, the refractive index of the dispersion media should be measured and included for the subsequent calculations of the sample diffusion coefficient.

The above protocol also highlights the minimum reporting requirements mentioned in ISO 13321:1996 (now ISO 22412:2017) that include: particle concentration (mass or volume based), dispersion medium composition, refractive index values for the particles and the dispersion medium, viscosity value for the medium, measurement temperature, filtration or other procedure used to remove extraneous particulates/dust prior to analysis (including pore size and filter type), cuvette type and size (path length), instrument make and model, scattering angle(s), and laser wavelength. Few research papers report all these values in the nanotechnology literature [47].

Over recent years, there have been significant efforts to uncover the reasons behind lack of reproducibility in the nanomedicine literature and to identify strategies to improve the robustness and accuracy of both characterization data and methodological approaches in nanomedicine. Aside from using checklists (e.g., MIRIBEL), the critical role of aforementioned standards is in addressing robust characterization of nanomedicines based on their intended use in medical devices and pharmaceuticals. The main issue with regard to the use of available standards in academic laboratories, however, is the challenge of “requiring” their implementations in the nanoscience literature. Peer reviewers, editors, and journal owners/publishers have historically seldom requested the details of standards or methods development in fundamental nanomaterials science, in the absence of biological applications [29], unlike standardization checklists often required for biological studies that mandate reporting of biological replicates, effect size calculations, ANOVA or other statistical significance calculations, antibody validation, plotting of all data points for N < 10 instead of averages, disclosure of error type (SD, SE, CI), and other standardized metrics by certain journals [48]. One critical challenge to similar standardization requirements for the nanomaterial and nano-bio interface literature is in the time lag between the inception of nanomaterials and nanotechnology as a relatively young field of study, and the time it takes to establish experimental and reporting standards. Another challenge is a lack of in-depth training expected or needed for undertaking studies in nanomedicine owing to the interdisciplinary nature of nanoscience. With multiple fields coalescing in nanobiotechnology, the experimental intuition and reproducibility standards that build from many years training in a specific field can be lacking. For example, using SEM and TEM for nanoscale imaging began as early as the 1930s according to the national nanotechnology initiative (NNI); however, the ISO standards related to characterizing nanomaterials by SEM (ISO 19749) and TEM (ISO 21363) were not published until 2021 and 2020, respectively. Similarly, recommendations by EUNCL and NCI-NCL are also relatively new, provided in the past ten years.

## Call to Action

It is well-documented that the nanomedicine literature suffers from poor reproducibility. In this paper, we have discussed one of the major causes of low reproducibility, stemming from overlooking available standard characterization methodologies, as the academic nanomedicine community may not fully aware of these standards.

Methods to characterize nanomaterials are complex, and various parameters can influence the accuracy and precision of the results. Although there is an urgent need to resolve the repeatability issue, there is no quick solution to this issue especially when assessing the magnitude and root causes of irreproducibility remain incomplete. Implementation of the discussed standards may seem to be a practical solution; however, the implementation process can be challenging considering the complexity and variability of methods associated with nanotechnology and therefore requires involvement of various stakeholders including research institutes, journal editors, and funding agencies. While we do not reiterate the technical details of the standardized protocols described herein, we emphasize the importance of incorporating these standards into research practice and communicating their importance with trainees in the nanoscience research community.

One technique highlighted here was DLS as it is the most commonly used instrument for nanoparticle size characterization in the literature. For so-called simple DLS measurements, many parameters can affect the reliability and repeatability of the measurement. Parameters such as particle concentration (mass or volume-based), dispersion medium composition such as salt concentration, refractive index values for the particles and the dispersion medium, viscosity value for the medium, measurement temperature, filtration, or other procedure used to remove extraneous particulates/dust prior to analysis (including pore size and filter type), cuvette type and size (pathlength), instrument make and model, scattering angle(s), and laser wavelength all can significantly affect the quality of the data from DLS. However, we could not identify manuscripts that had used DLS characterization that also reported these values and parameters, highlighting the issues at play that affect the ability to interpret DLS data and compromise the ability to reproduce the results in peer-reviewed literature.

## Conclusions

Herein, we have discussed the status of experimental validation standards and reproducibility in the nanoscience community, with implications for the translation of these nascent nanotechnologies into clinical practice. We hypothesize that one main contributing factor to the lack of rigor and reproducibility in nanoscience is due to different types of nanotechnologies, each of which is unique in its physiochemical and biocompatibility profiles and intended for a wide variety of downstream biomedical applications. We highlight that proper implementation and reporting of standards for characterization and methodology in nanomedicine need to be the subject of careful collaborative investigation and to be developed and broadly adopted by involved stakeholders, e.g., researchers, funding agencies, and journal editors, and provide examples of efforts toward these goals. Increasing awareness of the presence of established protocols and standards—and more importantly, implementing these standards in nanomedicine research—may improve the scientific rigor and reproducibility of nanoscience as applied to biomedicine and allow quantitative comparisons of results obtained by its various stakeholders.
